# Frequency of the virilising effects of attenuated androgens reported by women with hereditary angioedema

**DOI:** 10.1186/s13023-014-0205-6

**Published:** 2014-12-05

**Authors:** Zsuzsanna Zotter, Nóra Veszeli, Dorottya Csuka, Lilian Varga, Henriette Farkas

**Affiliations:** 3rd Department of Internal Medicine, Semmelweis University, Kútvölgyi út 4, Budapest, H-1125 Hungary

**Keywords:** Hereditary angioedema, C1 inhibitor deficiency, Attenuated androgen, Danazol, Virilization, Side effect

## Abstract

**Background:**

Danazol, a drug extensively used in the management of hereditary angioedema due to C1 inhibitor deficiency (C1-INH-HAE), has various side effects. This study investigated the virilizing actions of this drug in 31 danazol-treated female patients with HAE-C1-INH. We compared our findings with those of healthy controls and with literature data.

**Methods:**

The patients were interviewed individually about the type and severity of the virilizing effects, as well as about their satisfaction with danazol therapy.

**Results:**

The average duration of danazol treatment was 10.31 years [2 to 23] and its mean daily dose was 131.7 mg [33 to 200]. The most common adverse effects were hirsutism (n = 14), weight gain (n = 13), and menstrual disturbances (n = 8). The severity of danazol adverse effects did not differ by duration of treatment or by daily drug dose. The mean level of patient satisfaction with the treatment was high. The comparison of age-matched healthy controls and of HAE-C1-INH patients receiving danazol did not demonstrate a statistically higher incidence of any of the monitored symptoms in the danazol group.

**Conclusions:**

Our findings indicate that long-term danazol treatment – using the lowest effective dose – has only a mild virilizing effect.

## Background

Hereditary angioedema due to C1 inhibitor deficiency (C1-INH-HAE) is an autosomal dominant disorder; a form of bradykinin-mediated angioedema. It is characterized by recurrent, subcutaneous, and/or submucosal edema-formation. The clinical symptoms show inter- and intraindividual variation [1]. Women with C1-INH-HAE are more likely to experience symptoms than are men [2]. Female sex hormones – estrogens, in particular – affect the synthesis of many proteins influencing the activation of the kinin-kallikrein system [3,4]. The management of female patients with C1-INH-HAE is similar to that of males. It comprises two stages: *1)* the acute treatment of attacks, and *2)* prophylaxis [3]. As regards medicinal products for long-term prophylaxis (such as antifibrinolytics, attenuated androgens [AAs], and plasma-derived C1-INH), the use of attenuated androgens in women with HAE raises a number of concerns [5]. Although the precise mode of action of AAs is unknown, these agents are thought to increase C1-INH level by stimulating hepatic synthesis, as well as by enhancing the expression of C1-INH mRNA in peripheral blood mononuclear cells [6]. The AAs also may have a potentiating effect on Aminopeptidase P, which is involved in kinin inactivation [7]. While AAs are not intended for the treatment of C1-INH-HAE, these drugs have found their way into clinical practice through empirical use. AAs are effective as prophylaxis to reduce the frequency and severity of edematous attacks in C1-INH-HAE [8]; however, their benefits should be weighed against their risks before prescription. Major side effects of AAs include hepatotoxicity, altered/pro-atherogenic lipid profile, stimulated erythropoesis, androgen-induced premature closure of the epiphyseal plates in children, and a virilizing/androgen-like effect [9]. The latter is the primary concern in the management of female patients. Reviewing the literature, we found only a few studies evaluating the virilizing/androgen-like effects of AAs in female patients with C1-INH-HAE [10-14]. These studies did not explore systematically the whole range of virilizing side effects, and even those that did, failed to analyze male and female patients separately – or lacked a control group. Therefore, we investigated and evaluated the frequency and severity of the possible virilizing effects of AAs in female patients with C1-INH-HAE, as well as compared our findings with the results of healthy controls and with literature data. Our study did not analyze either therapeutic efficacy, or the spectrum of other adverse events (such as altered lipid profile, hepatotoxicity, etc.), as we have published papers on these topics earlier [15-17].

## Patients and methods

### Patients

All (31 of our 75) female patients with C1-INH-HAE (median age: 49 years; min: 20 max: 70), who were taking danazol for long-term prophylaxis participated in this survey. All subjects had been diagnosed and receiving regular follow-up care at the Hungarian Angioedema Center. In each patient, C1-INH-HAE was diagnosed according to the accepted clinical and laboratory criteria (positive family history, clinical symptoms of angioedema, low functional C1-INH level, low C4, normal C1q) [1]. At the time of the study, 16 patients with C1-INH-HAE were postmenopausal, one patient was taking an oral contraceptive (desogestrel).

After diagnosis, the patients attended follow-up visits at least once a year. A laboratory screen (including complete blood count, clinical chemistry, serum lipid profile, and liver function), abdominal sonography, and physical examination were performed on these occasions. Body weight was recorded along with the current drug regimen and dose administered, as well as the side effects experienced by the patients.

The control group consisted of 41 healthy and age- matched female subjects (median age: 48 years; min: 32 max: 63). 22 of 41 were postmenopausal, 9 of them longer than 5 years, and 7 women were taking an estrogen-containing oral contraceptive. All had been referred for routine medical check-up, and volunteered for the study by giving informed consent. The healthy controls did not have any known disease (C1-INH deficiency was excluded by complement testing), or receive medicinal products at the time of blood sampling.

## Methods

In our retrospective study, we compiled a list of the virilizing adverse effects of danazol by interviewing the patients, and reviewing the literature. We identified the following: menstrual disturbances (spotting, disruption of the menstrual cycle), altered libido, voice change, body hair growth (evaluated in 7 body regions by self-examination: upper lip, chin, chest, back, abdomen, thighs and legs), hypertrophy of the clitoris, increased muscle mass, reduction of breast size, acne and oily skin, weight gain, water retention, hair loss, psychological abnormalities (depression, aggressive behavior), flushing, sweating. At the follow-up visits, we provided patients with the list of all possible virilizing side effects and asked them to mark every symptom or sequel they had experienced during danazol therapy and rate their severity using the following scores: 0 = none, 1 = mild, 2 = moderate, 3 = severe. Furthermore, the patients were asked to rate the overall severity of the adverse effects (considering all types aggregately). Patient satisfaction with danazol therapy (weighing adverse effects against benefits) was rated on a Visual Analog Scale (VAS; 100 mm per scale). The daily dose of danazol was determined as the mean of the daily doses received during the course of therapy. During prophylaxis, we placed emphasis on administering the lowest effective dose. The 41 age-matched, healthy controls also completed the survey on the adverse events they had experienced during the last five years. The study was approved by the institutional review board of Semmelweis University of Budapest, and informed consent was obtained in accordance with the Declaration of Helsinki.

We used GraphPad Prism 6 software for statistical analysis. Spearman’s correlation tests were applied to evaluate correlations. Chi-squared statistics were used to detect any significant difference among the study parameters of the HAE patients and those of the healthy controls. All the statistical analyses were two-tailed, and p < 0.05 was considered to represent a significant difference, or correlation.

## Results and discussion

Thirty-one female patients with C1-INH-HAE took danazol for long-term prophylaxis. The average dose was 131.7 (min: 50, max: 200) mg/day. The mean duration of pre-existing danazol treatment was 10.31 (min.: 2, max.: 23) years.

**Figure 1:**
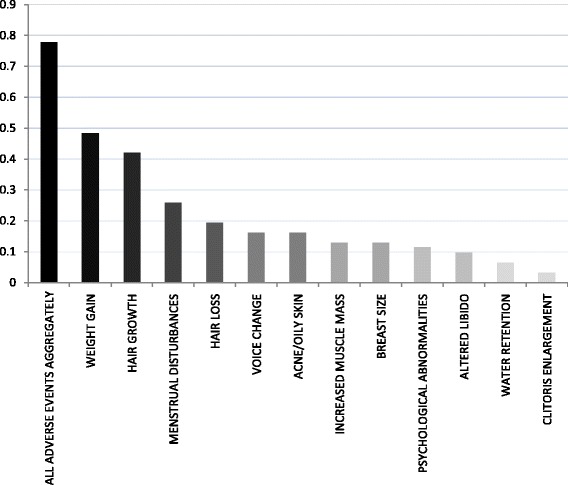
**The severity scores of each and all adverse events, ranked by severity on a 3-grade scale.**

The patients experienced on average 2.9 out of the 12 monitored virilizing adverse effects. Five patients did not experience any side effect whatsoever. Hirsutism (n = 14, 42%), weight gain (n = 13, 39%, 10 kilograms on average), and menstrual disturbances (n = 8, 26%) were the virilizing effects most commonly noted by the C1-INH-HAE patients. Table 1 shows our results in comparison with literature data, whereas Figure 1 presents the mean severity scores of the individual adverse effects. The patients rated the majority of these as mild (0 or 1). One patient assigned a score of 3 to weight gain experienced as an adverse effect, and two additional patients rated hair growth and menstrual disturbances with a score of 2.

**Table 1:** **Comparison of the virilizing adverse effects of AAs among different studies**

	**Gelfand [** 7 **]**	**Zurlo [** 8 **]**	**Cicardi [** 9 **]**	**Sloane [** 10 **]**	**Bork [** 11 **]**	**Our study population**	
						***Patients***	***Controls***
N° of patients	5	60	28 (D + S)	13 (S)	51/118*	31	41
MENSTRUAL DISTURBANCES	100%	79%	50% (D)/ 18% (S)	23%	76%	26%	38%
ALTERED LIBIDO		10%		8%	3%*	13%	22%
VOICE CHANGE		8%		8%	14%*	19%	10%
HAIR GROWTH		12%			19*	42%	37%
CLITORIS ENLARGEMENT					12%	3%	0%
INCREASED MUSCLE MASS		2%			3%*	13%	7%
BREAST SIZE					12%	16%	n.a.^#^
ACNE/OILY SKIN		8%		8%	9*	19%	12%
WEIGHT GAIN	100%	60%	28% (D)/17% (S)	1%	42%*	39%	59%
WATER RETENTION		2%				6%	29%^†^
HAIR LOSS		18%				19%	27%
PSYCHOLOGICAL ABNORMALITIES		2%		0%	11%*	16%	46%^†^
Discontinuation/continuation of danazol therapy	Continued	16% discontinued	The vast majority of patients continued therapy	No interruption of stanozolol therapy	No interruption of stanozolol therapy	No interruption of danazol therapy	

Although we would have preferred specifying the absolute numbers of patients, this was not feasible, because not all the authors provided this information. Therefore, all values are expressed as percentages, for the sake of comparability. References are reported in brackets.

D = danazol, S = stanazolol, * = total HAE population (The properties were evaluated both in males and in females), ^†^ = p < 0.05, ^#^n.a = Half of the women did not answer this one, or gave information on the elasticity of their breasts.

The mean score reflecting the overall severity of the side effects was 0.7 (min.: 0, max.: 3; see Figure 1). The Spearman’s test did not reveal a significant correlation between the overall severity score of adverse effects and the duration of treatment (p = 0.599), or the daily dose of danazol (p = 0.204).

Analyzing the most common and most severe adverse effect separately with Spearman’s test, we could not detect any significant correlation between the patients’ BMI and danazol dose (p = 0.671). We found a significant, positive relationship between the patients’ BMI and the duration of treatment (p = 0.031). The change in body weight did not correlate with either the dose (p = 0.818), or with the duration of danazol treatment. Menstrual disturbances occurred in 8 patients. Six of these patients discontinued danazol (because they wanted to have a child) and their menstrual cycles became regular again.

The mean score (VAS, 100 mm per scale) of patient satisfaction was 84 (min.: 30, max.: 100). None of the patients discontinued danazol treatment due to virilizing adverse events. The Pearson’s test did not detect any significant correlation between patient satisfaction with danazol and the duration of treatment, or the daily drug dose.

Comparing female HAE patients with their age-matched, healthy controls, we did not detect a statistically significant difference in the majority of the study parameters. Water retention and psychological abnormalities were exceptions, as their incidence was statistically higher in the control group than among the patients (29% *vs*. 6%, p = 0.0177; and 46% *vs*. 16%, p = 0.011, respectively).

Our findings indicate that long-term treatment with the lowest effective dose of danazol has only a mild virilizing effect, because the patients experienced three on average out of the 12 monitored adverse events. The severity of the virilizing adverse effects (scored in the range from 0 to 3) was below 0.5 in all instances, and the overall score was as low as 0.7 for the whole study population. Of note, the mean age of our patients was in the age range when menopause usually occurs – thus, these adverse effects might even have resulted from spontaneous pro-androgenic events. In agreement with literature data, the most common adverse effects included hirsutism, weight gain, and menstrual disturbances (Table 1). Weight gain has not yet been demonstrated to be a direct consequence of treatment with AAs, although we found that the longer the duration of treatment, the higher the body mass index of the female patients. However, the magnitude of the change in body weight did not correlate with any of the above parameters. Hirsutism occurred more commonly than in the other studies, but its frequency did not exceed that seen in the Hungarian general population, as well as it was of mild severity. Menstruation disturbances were common but reversible and resolved after AAs had been discontinued; this is also in agreement with the observations published in the literature.

Many authors emphasized that these adverse effects are dose-related [14,18,19]. We could not demonstrate this relationship in our study, because our patients were taking relatively low doses. Nevertheless, this observation further supports the practice of administering the lowest effective dose to prevent side effects. Discontinuing prophylactic treatment because of the virilizing effect of danazol was not necessary in any of our patients. This matches the experience of other authors, who have found that only a small proportion of patients had to stop taking danazol due to its virilizing effect [12-14].

The reports available on the virilizing adverse effects of danazol indicate that these events commonly occur in women taking this medicinal product. However, this does not necessarily mean that these side effects are severe. In our study population, the severity scores of the adverse events consistently remained below the ‘mild’ rating – possibly, because we had administered average or lower doses. To date, the studies published in the literature reported the incidence of adverse events without analyzing their severity. This might explain the incongruity between the abundance of virilizing adverse effects attributed to danazol, and the small number of patients who discontinue treatment because of these events (mostly those receiving higher doses). Further, a causal relationship between treatment with danazol and the occurrence of virilizing adverse effects cannot be established with certainty, because the difference compared with the healthy population was not statistically significant. In view of the patients’ age, these symptoms might even be attributable to the menopause.

## Conclusion

The virilizing effects of AAs are mild at the lowest effective dose not to exceed 200 mg. State-of-the-art medicinal products with known mechanisms of action, as well as with confirmed efficacy and safety have become available (such as plasma-derived nanofiltered C1-INH concentrate – Cinryze) [20]. Other alternatives (for example recombinant human C1-INH) for prophylaxis are also under investigation [21]. Notwithstanding this, treatment with attenuated androgens is considered an alternative for prophylaxis. These agents are administered by the oral route, which is convenient for the patients and economical for the health service provider. The occurrence of adverse effects can be prevented or their timely recognition is feasible through regular follow-up at least once a year [22,23]. Further, novel therapeutic options are not yet available in many countries. Therefore, abandoning treatment with AAs is not yet possible, because in many countries, these drugs offer the only therapeutic option for severe cases.

## Abbreviations

HAE: Hereditary angioedema
C1-INH: C1-Inhibitor
C1-INH-HAE: Hereditary angioedema due to C1-inhibitor deficiency
AAs: Attenuated androgens
VAS: Visual analogue scale
